# A Newly Developed Web-Based Resource on Genetic Eye Disorders for Users With Visual Impairment (Gene.Vision): Usability Study

**DOI:** 10.2196/19151

**Published:** 2021-01-20

**Authors:** Jian Lee Yeong, Peter Thomas, James Buller, Mariya Moosajee

**Affiliations:** 1 Institute of Ophthalmology University College London London United Kingdom; 2 Moorfields Eye Hospital NHS Foundation Trust London United Kingdom; 3 Aniridia Network Sheffield United Kingdom; 4 Great Ormond Street Hospital for Children NHS Foundation Trust London United Kingdom

**Keywords:** internet access, blindness, eye disease, genetic diseases, usability testing, qualitative research, internet-based intervention, consumer health information, mobile phone

## Abstract

**Background:**

Despite the introduction of the Web Content Accessibility Guidelines and legislations, many websites remain poorly accessible to users with disability, especially those with visual impairment, as the internet has become a more visually complex environment. With increasing reliance on the internet and almost 2 million people in the United Kingdom being affected by vision loss, it is important that they are not overlooked when developing web-based materials. A significant proportion of those affected have irreversible vision loss due to rare genetic eye disorders, and many of them use the internet as a primary source of information for their conditions. However, access to high-quality web-based health information with an inclusive design remains a challenge for many. We have developed a new web-based resource for genetic eye disorders called Gene.Vision that aims to provide a holistic guide for patients, relatives, and health care professionals.

**Objective:**

Through a usability testing session of our website prototype, this study aims to identify key web-based accessibility features for internet users with vision impairment and to explore whether the contents provided in Gene.Vision are relevant and comprehensible.

**Methods:**

A face-to-face testing session with 8 participants (5 patients, 2 family members, and 1 member of the public) and 8 facilitators was conducted on a prototype website. Remote testing was performed with another patient due to COVID-19 restrictions. Home page design, navigation, content layout and quality, language, and readability were explored through direct observation and task completion using the think-aloud method. A patient focus group was organized to elicit further feedback. Qualitative data were gathered and analyzed to identify core themes through open and axial coding.

**Results:**

All participants had good computer literacy; 6 patients with visual impairment used visual aid software including iOS VoiceOver and Speak Screen, iOS Classic Invert, ZoomText 2020, Job Access With Speech, and Nonvisual Desktop Access. The features identified by the participants that will enhance accessibility and usability for users with visual impairment were a consistent website layout, a structured information hierarchy with a clear description of links, good chromatic and luminance contrast, a simple home page with predictable and easy navigation, adaptability to various assistive software, and readable and relevant content. They reported that dynamic content (such as carousels) and large empty spaces reduced accessibility. Information on research, support available, practical advice, and links to charities were incentives for repeated website visits.

**Conclusions:**

We demonstrated the importance of developing a website with a user-based approach. Through end user testing, we identified several key web-based accessibility features for people with visual impairment. Target end users should always be involved early and throughout the design process to ensure their needs are met. Many of these steps can be implemented easily and will aid in search engine optimization.

## Introduction

### Background

People with disabilities have been recognized to experience inequalities in multiple aspects of their lives from education and employment to health care, finances, and leisure [1], with the internet being a key accessibility feature in all areas. It was intended to be user-friendly for every individual regardless of any mental or physical disability but poor design and coding of what is usually thought of as a graphical user interface poses difficulties, particularly for users with visual impairment [2-4]. With an increasing reliance on the internet and almost 2 million people in the United Kingdom being affected by vision loss, of which 360,000 are registered with visual impairment or severe visual impairment (blind), it is important that they are not overlooked when developing web-based materials lest they risk being socially excluded altogether [5,6].

To make the internet more accessible, the World Wide Web Consortium first introduced the Web Content Accessibility Guidelines (WCAG) in 1999; its current iteration (WCAG 2.1), published in 2018, serves as an update rather than as a replacement of WCAG 2.0 (introduced in 2008). It contains 13 guidelines based on 4 main principles: perceivable, operable, understandable, and robust, with each guideline having 3 levels of testable success criteria—A (lowest level of accessibility) and AA and AAA (highest level of accessibility) [7]. Both the United Kingdom and the European Union have adopted it into their legislation, making it a legal requirement for public sector websites to be accessible to people with disabilities [8,9].

Despite this, only a small number of websites are compliant with the UK accessibility standard of WCAG 2.1 AA [10]. With consumers increasingly turning to the internet as a primary source of health-related information, websites should not only contain relevant and helpful information for patients but should also not exclude anyone with a disability. To help developers of web-based health platforms achieve this, the UK National Health Service (NHS) published a Digital Service Manual focusing on building consistent, usable, and accessible services using a patient-centric approach [11]. It provides guidance on various aspects of web design, such as conducting user research, building user interface prototypes, content styles, and optimizing accessibility.

A significant group of web-based health information consumers are those affected by rare diseases that together affect 1 in 17 people [12]. Owing to the nature of their conditions, their physicians often lack the experience or familiarity to provide expert care for them. They end up searching the web to keep abreast of current research and look for support through online communities. However, the quality of these websites tends to be poor, either they lack credibility or the contents are not kept up-to-date or pitched at the right level [13]. Furthermore, most of these sites are found to be poorly accessible to users with visual impairment, which can have a profound impact on patients affected by rare genetic eye disorders who usually have visual disability [13,14]. Inherited retinal diseases are the most common causes of severe visual impairment registration among working adults in England and Wales, whereas globally, 60% of blindness among infants is due to genetic diseases [15,16].

### Objectives

To fill this void, we are developing a new web-based resource for these conditions with information written in 2 formats: one using more lay terms for patients and families, while the other has more technical details tailored toward health care professionals. It aims to provide a holistic resource, including the causative gene or genes of a condition, current research, management, and support services. Most importantly, we prioritized creating a usable and highly accessible website for our target end users, who mainly have visual impairment.

Although automated testing tools can help identify some of the website’s accessibility barriers, most of the WCAG 2.1 guidelines require human testing and judgment. Failing to trial a specific design with users with disability will result in a website with limited *usable accessibility*, as the human interface aspect is not evaluated [17]. Therefore, the NHS Digital Service Manual recommends that research and testing with real-world users are performed early and throughout the design process [18]. In line with this recommendation, we conducted a usability testing session on a prototype of our website with patients to identify website features that would increase accessibility for users with visual impairment. Our secondary objective is to explore whether the contents provided on our website were relevant and comprehensible to both patients and clinicians.

## Methods

### Prototype

Before development, we spoke to patients with genetic eye disorders and their families to identify potential *pain points*, which were websites with poor accessibility, unreliable and outdated information, contents written with excess medical jargon and insufficient material on research, practical advice, and available support. An accessibility specialist, who was a patient herself (see the Acknowledgments section), was also consulted to identify key accessibility features for those with visual impairment. On the basis of feedback from these interactions, we built a prototype of our website using the Wikimedia platform (Wikimedia Foundation, Inc) [19], which contained the following components.

#### Home Page

**Figure 1:**
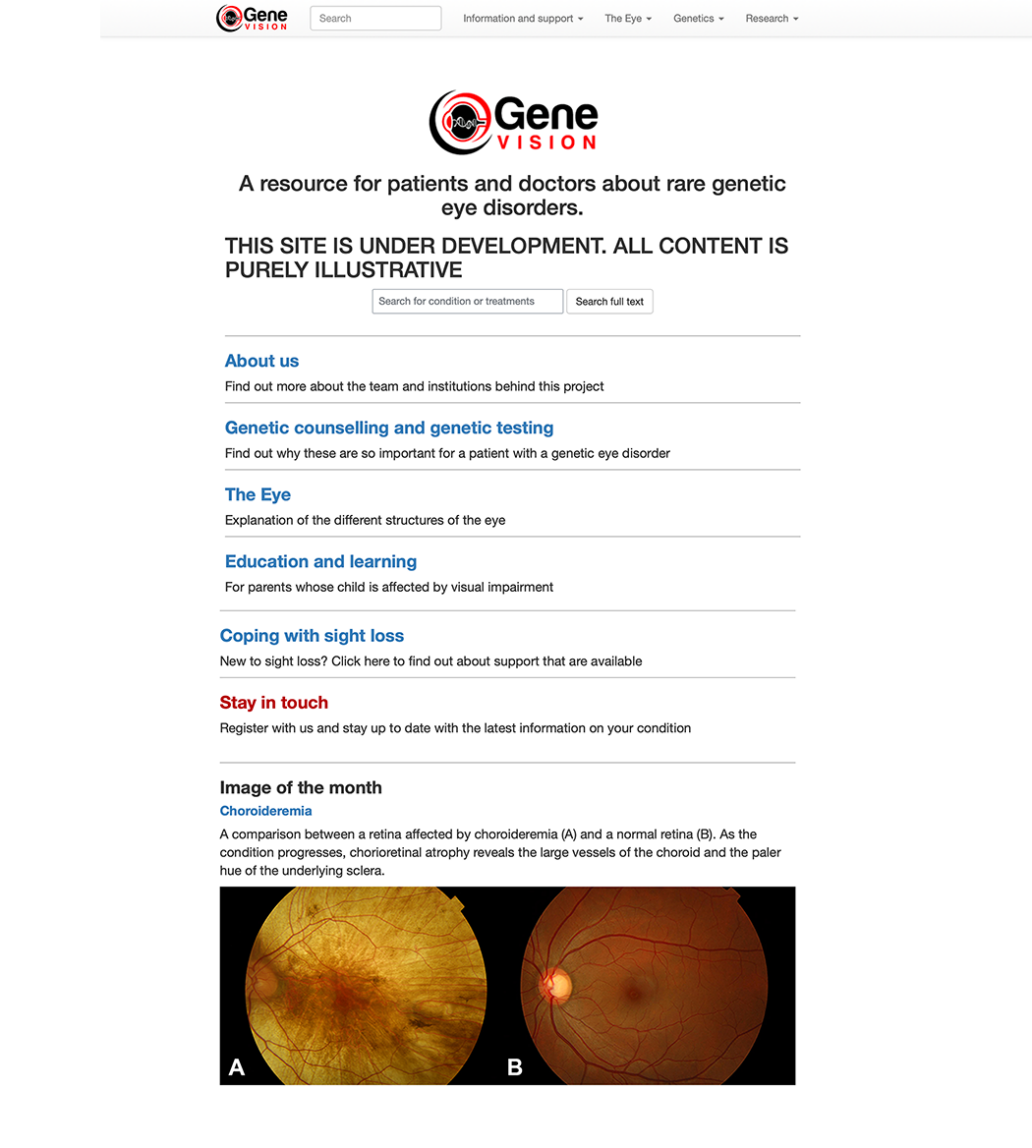
Home page of the Gene.Vision Wikimedia prototype. The navigation bar serves as the header of the page. The search box is above the listed menu items. Our participants pointed out that the navigation bar and search box had poor chromatic and luminance contrast. The large area of white space on the right in portrait mode can disorientate magnifier users.

**Figure 2:**
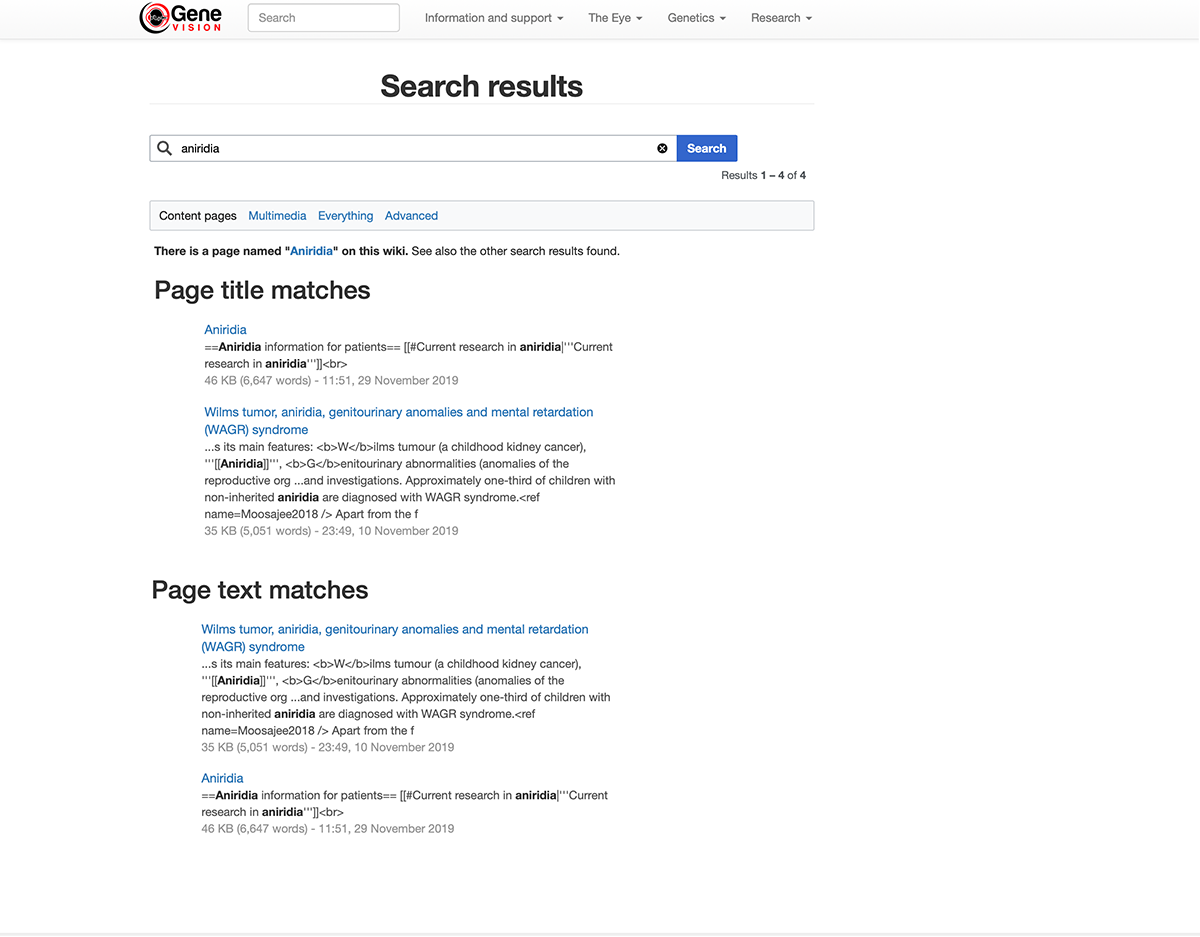
Search results for the term aniridia. Our participants found the display confusing compared to what they are used to from popular search engines.

The home page’s navigation bar is organized into 4 main items: information and support, the eye, genetics, and research (Figure 1). The subcategories of each main item are listed in Textbox 1 along with a description for each category. There is a search box in the center whereupon a search term or terms is entered, all pages containing the term or terms are displayed on a new page (Figure 2).

**Subcategories of each main item on the navigation bar in the Gene.Vision prototype with a brief explanation of each category.**

Information and support

- Coping with sight loss: outlining support services that are available for patients recently diagnosed with vision loss. It also explains the roles of low vision clinic and eye clinic liaison officers and directs patients to resources on assistive technology
- Registration for sight impairment: the process of certification and registration are outlined here along with the associated advantages
- Driving: the minimum driving standards in the United Kingdom are explained here with further links to the government information portal. Patients are also signposted to alternative ways of transportation if they are no longer able to drive
- Education and learning: the various supports that are available for children with visual impairment are listed here with advice on how to access these services
- Charles Bonnet Syndrome (CBS): an explanation about CBS, coping mechanisms, current research and links to organizations offering support to those affected

The Eye (pages providing lay explanations of the following ocular structures):

- Conjunctiva
- Sclera
- Cornea
- Trabecular meshwork
- Iris and pupil
- Lens
- Vitreous humor
- Retina
- Optic nerve
- Choroid

Genetics

- Introduction to genetics: a brief overview of DNA, genes, and chromosomes
- Inheritance patterns: explanations on the more common inheritance patterns encountered in genetic eye disorders (autosomal dominant, autosomal recessive, X-linked, and mitochondrial)
- Genetic counseling and genetic testing: the role of genetic counselors, the process of genetic testing and its associated benefits and limitations are explained in this page

Research

- Clinical trials: explaining the different aspects of clinical trials including the various phases, the role of ethics committees, and the benefits of participation
- Treatments under research: this section contains various investigative therapies for genetic eye disorders with links to their corresponding pages. Only gene therapy was prepared in this prototype
- Participate in research: This link aims to connect to a website listing all the current ongoing trials in Moorfields Eye Hospital. This website has not been set up yet during the testing session nor at the time of writing this manuscript

To highlight certain relevant topics to users, some of the subcategories from the main navigation bar were placed into a list below the search box, each having a brief excerpt (Figure 1).

In addition to the Wikimedia-based home page, we also designed another home page prototype using WordPress for the purpose of this testing. The screen recordings of both home page designs are available in Multimedia Appendices 1 and 2.

#### Content Pages

##### Conditions

**Figure 3:**
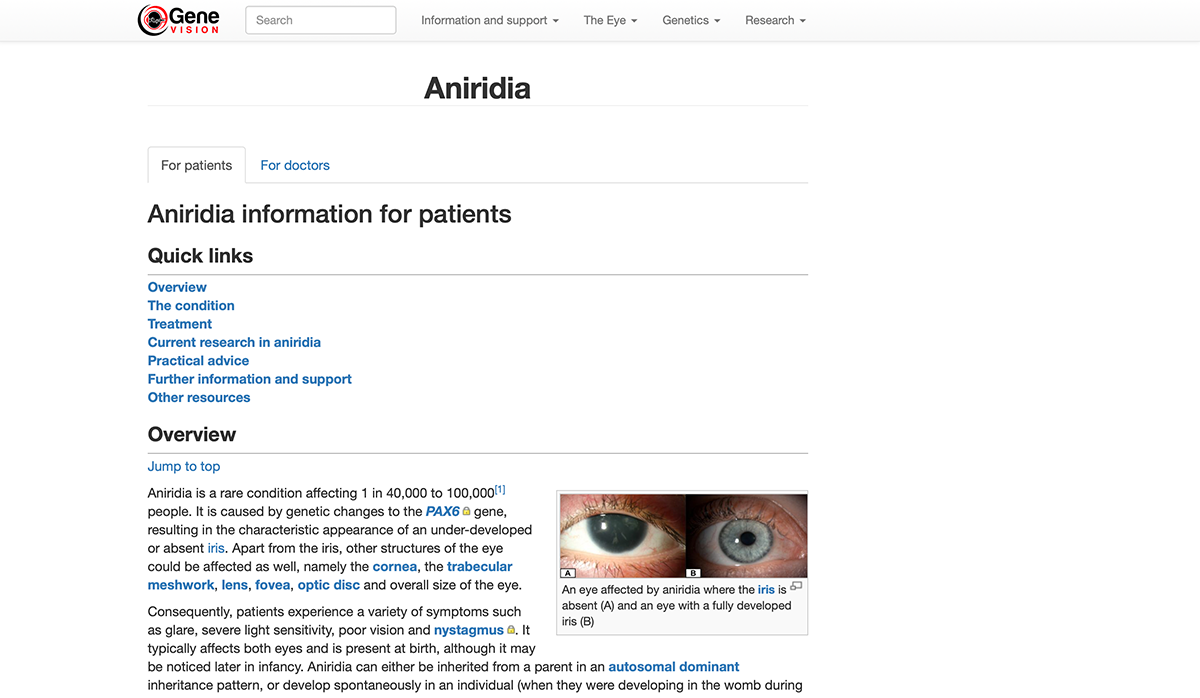
Example of a condition page. Users can toggle between the patient and health care professional versions using the For patients and For doctors tabs. A table of contents is placed under the Quick links section, where users can go directly to the topic of interest without additional scrolling. Jump-to-top links are available under every heading to facilitate quick navigation back to the Quick links section.

Two versions are available for each condition page: one for the patients and general public, and the other for health care professionals. Users can toggle between these 2 versions using the tabs below the main page title (Figure 3).

The former focuses on aspects that we believe patients will find helpful, such as current research, information on available support, and links to both umbrella and condition-specific charities. When possible, we asked a patient representative from a charity related to the condition to review our material for readability. The health care professional version focuses more on the clinical presentation, investigations, general management guidance including genetic counseling, genetic testing, and updates on current research. Full citations are listed in both versions.

A total of 3 conditions were prepared for this prototype: choroideremia; aniridia; and Wilms tumor, aniridia, genitourinary anomalies, and range of developmental delay (WAGR) syndrome. The patient versions of the choroideremia and aniridia pages were reviewed by patient representatives from Retina UK and Aniridia Network, respectively. The review of the WAGR syndrome page was still pending at the time of testing.

##### Other Pages

For pages listed in Textbox 1, only the patient or general public version was available in the prototype.

The pages that were prepared for this prototype were as follows: *Coping with sight loss*, *Driving*, all the pages listed under *The eye, Genetic counseling and genetic testing, Clinical trials*, and *Gene therapy*.

To improve user navigation, a table of contents is listed at the top of each page termed *Quick links* (Figure 3). Users are able to navigate to each subheading by clicking on the topic of interest. They can easily navigate back to *Quick links,* by using the *Jump to top* link. A screen recording of this function is available in Multimedia Appendix 3. Each page always starts with a brief overview of the selected topic, with the structure and font used consistently throughout the website. The information provided on all pages is based on current UK practices. All images on the website were accompanied with the alt-text function, except those that were for decorative purposes only. The prototype was tested manually with the NonVisual Desktop Access screen reader (NV Access) by one of the authors (JY) before the usability testing session to ensure compatibility and troubleshoot any fundamental accessibility barriers (eg, unclear alt-text, missing or ambiguous links).

#### Usability Testing

Participants were recruited through the vision loss charity Retina UK and from the senior author’s (MM) genetic eye disease clinic in Moorfields Eye Hospital NHS Foundation Trust, London.

A face-to-face usability testing session and patient focus group (1 participant to 1 facilitator) of our prototype was conducted at the University College of London Institute of Ophthalmology computer laboratory on November 11, 2019. Participants were encouraged to bring their personal devices that they usually used to access the web. For those who chose to use the laboratory’s Windows PC desktops, assistive software was installed upon prior request. They also chose which web browser to use based on their experience and comfort level. The device, web browser, and assistive software used to access the website were recorded by the facilitators.

A short survey (Multimedia Appendix 4) was conducted at the beginning of the session to gather information about the demographics and computer literacy of each participant. This was assessed using a 5-point Likert scale based on the UK government digital inclusion scale [20]:

- Not at all confident: someone who does not know how to use the internet at all.
- Slightly confident: able to perform tasks on the internet with full guidance.
- Somewhat confident: able to perform certain tasks independently, does not tend to deviate from these tasks when using the internet (task-specific).
- Very confident: someone considered to have basic digital skills and able to use the internet effectively.
- Extremely confident: someone who usually has a background in computing or is able to code or design content.

The last 2 questions of the survey focused on the participant’s current source of information about their condition and the type of information they considered important but currently lacking.

During the testing session, we used 2 usability techniques: direct observation and task completion. The tasks outlined for the participants (Textbox 2) were designed to identify specific features and issues pertaining to the home page design, site navigation, and content. For our condition pages targeted to the patients and general public, we used identical pages from Wikipedia as a benchmark because it is one of the most viewed medical resources globally and tends to appear as one of the top results in general search engines for keywords associated with rare diseases compared with other web-based health resources [21,22]. Furthermore, Wikipedia has a standardized information structure optimized for accessibility (WACG 2.0) and contributors are encouraged to follow the guidance provided in their *Manual of Style* to maintain this standard [23].

**The tasks set out for the participants of the usability testing session.**

Home page

1. Home page design: Participants were asked to compare the 2 home page designs, Wikimedia (Wikimedia Foundation, Inc) and WordPress, and highlight the aspects that improved or affected accessibility for each design
2. Navigation: navigating to the *Driving, Retina,* and *Genetic counselling* and *genetic testing* pages by using a combination of the navigation bar, search box, and the listed home page menu. While testing the navigation bar, we wanted to find out if participants preferred a drop-down menu displaying the full list of items (eg, *Information and Support* section) or a tab that links to a separate page containing the full list of items (eg, *The Eye* section). Participants were asked to explore each of the assigned pages and assess the relevance and readability of the content provided

Condition pages

- Participants were required to read 2 condition pages on our website: choroideremia and aniridia. They were asked to compare identical pages from Wikipedia with regards to information layout, readability, and quality of content. They then had to point out features that they liked and disliked from both the prototype and Wikipedia

While performing the assigned tasks, participants were encouraged to verbalize their initial impressions of the website, its accessibility and ease of use, comprehensibility of the content, and suggestions for improvement (think-aloud method). They were prompted by the facilitators to explain their actions and expectations. All responses were recorded by each facilitator in a standardized proforma. The facilitators primarily served as passive observers (direct observation) and were only allowed to assist if their respective participant reached an obstacle, at the same time noting how the participant encountered this.

A patient focus group for all the participants was conducted by the main facilitator at the end of the usability testing session to encourage feedback and discussion. Participants were asked about their general thoughts about the website, whether the current version was usable by individuals with visual impairment, any features that they liked or disliked, and if there were any changes or additions they would like to see. Further discussions were prompted by asking participants to expand their answers and seek opinions or counter-opinions from the rest of the group. The focus group was audio taped and transcribed after obtaining informed consent from all participants. A summary of the discussions was prepared after recording by the main author (JY).

#### Clinician Testing

The website was also created as a resource for health care professionals who may see patients with genetic eye diseases and require further information on the condition and management advice. Hence, the content was assessed remotely by a range of health care professionals from various disciplines, including a consultant general practitioner, a consultant pediatrician, 2 ophthalmology trainees, 3 ophthalmology consultants of different subspecialties (general, pediatrics, and medical retina), and a genetic counselor. Their assigned tasks were similar to those outlined in Textbox 2, with particular focus on home page navigation, information layout, and content quality in the professional versions of choroideremia, aniridia, and WAGR syndrome. Feedback was recorded in the same proforma used in the usability testing session.

#### Qualitative Data Analysis

The data collected in the standardized proformas and the audio transcripts from the patient focus group were analyzed by one author (JY) in an inductive manner rather than theory based, as we wanted to understand participants’ experiences with the prototype. Codes were attributed to various key phrases and paragraphs in both the written and audio transcripts (open coding) to identify core themes. Axial coding was then applied to develop connections between the open codes. Coding was performed with the qualitative software program NVivo 12 (QSR International).

## Results

### Participant Characteristics

There were 8 participants in our usability testing session and their demographics are presented in Table 1. A participant (P9), who used the popular Job Access With Speech (JAWS) screen reader, tested the website remotely during the COVID-19 lockdown period in the United Kingdom. In total, 6 participants were affected by genetic eye disorders, while 2 were parents of a patient with type II Usher syndrome. One participant had full sight and worked as an information specialist in a library. Most participants had basic digital skills, but 3 participants were considered experts. One participant considered herself a *task-specific* web user. The devices, web browsers, and assistive software used by the participants are shown in Table 2. The full results of the pretesting survey (including P9) are presented in Multimedia Appendix 5.

**Table 1:** Demographics of the participants in the usability testing session.

Participant	Gender	Age (years)	Employment status	Internet usage	Confidence in using the internet	Health status	Diagnosis
P1	M^a^	53	Employed	Yes	Very confident	Unaffected parent	Son-type II Usher syndrome
P2	F^b^	52	Employed	Yes	Very confident	Unaffected parent	Son-type II Usher syndrome
P3	F	41	Unemployed	Yes	Somewhat confident	Patient	Retinitis pigmentosa
P4	F	62	Retired	Yes	Very confident	Patient	Cone-rod dystrophy
P5	M	40	Employed	Yes	Extremely confident	Patient	Aniridia
P6	M	37	Employed	Yes	Very confident	Patient	Retinitis pigmentosa
P7	F	25	Employed	Yes	Extremely confident	Patient	Type II Usher syndrome
P8	M	45	Employed	Yes	Extremely confident	Unaffected individual	N/A^c^
P9^d^	M	70	Retired	Yes	Very confident	Patient	Cone-rod dystrophy

^a^M: Male.

^b^F: Female.

^c^N/A: not applicable.

^d^P9 was only able to test the website remotely due to COVID-19 lockdown rules.

**Table 2:** Device, web browser, and assistive software used by each participant.

Participant	Device	Web browser	Assistive software
P1	Windows PC	Google Chrome (Google LLC)	N/A^a^
P2	Windows PC	Google Chrome (Google LLC)	N/A
P3	iPhone	Safari (Apple Inc)	iOS VoiceOver (Apple Inc)
P4	Windows PC	Mozilla Firefox (Mozilla Foundation)	Zoomtext 2020 (Freedom Scientific) with invert color setting
P5	Windows PC	Google Chrome (Google LLC)	Windows magnifier at 200% magnification (Microsoft)
P6	iPad	Safari (Apple Inc)	iOS Classic Invert, iOS VoiceOver (Apple Inc)
P7	iPad	Safari (Apple Inc)	iOS Large Text, color filters, iOS VoiceOver and Speak Screen (Apple Inc)
P7	Windows PC	Google Chrome (Google LLC)	NVDA^b^
P8	Windows PC	Google Chrome (Google LLC)	N/A
P9	Windows PC	Google Chrome (Google LLC)	JAWS^c^

^a^N/A: not applicable.

^b^NVDA: NonVisual Desktop Access.

^c^JAWS: Job Access With Speech.

### Qualitative Analysis

#### Participant Feedback

**Figure 4:**
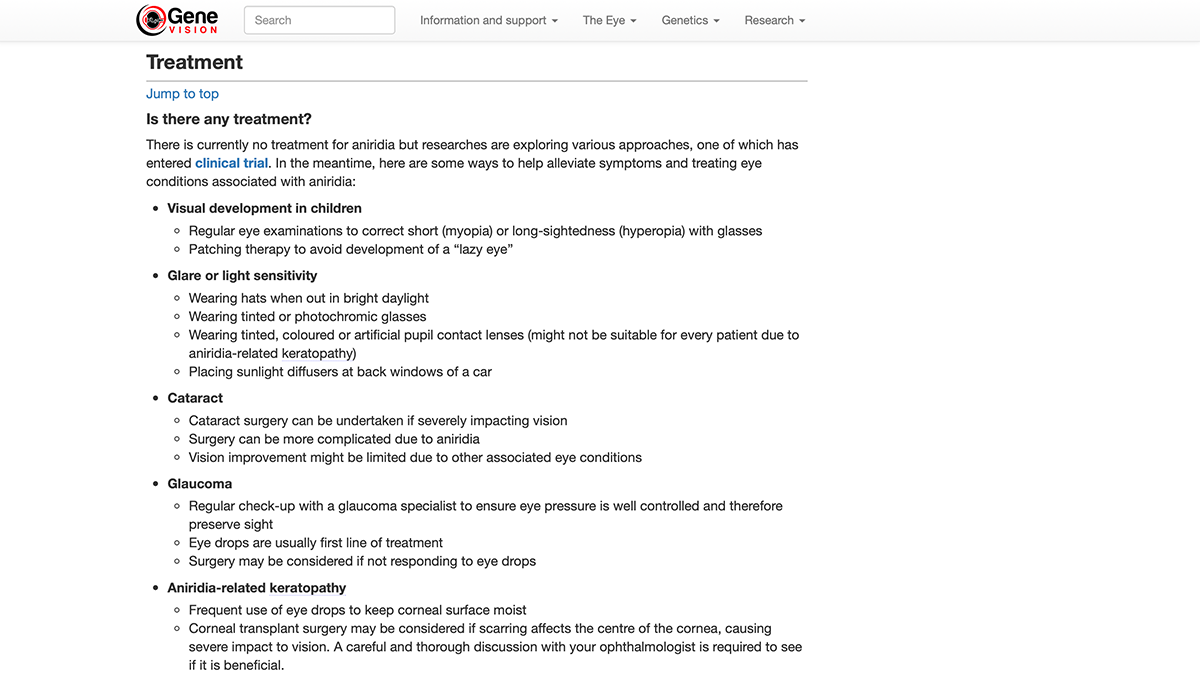
Example of content arrangement using bullet points for ease of reading. The clinical trial link is not underlined here, which may confuse users with sight impairment.

The positive features of the website that enhanced accessibility for users with visual impairment are outlined in Textbox 3. One of the main features highlighted by the participants was the consistent layout throughout the website. These included having left-aligned texts, using a consistent font and color along with having the Quick links section in all pages. They identified that having a zigzag alignment of text, as in the case of the WordPress-designed home page (Multimedia Appendix 2), limited accessibility. For the content pages, our participants noted that having clear and concise information helped with accessibility. These included having accurate and logically ordered headings, arranging information using bullet points for ease of reading (Figure 4) and the purpose of each link clearly identified from the link text alone. Another important aspect that favored accessibility was good adaptability to various assistive software and devices, especially in this day and age where mobile devices are increasingly popular and accessible. The participants also favored a simple home page that was easy to navigate to other pages on the website. Participants with and without visual impairment liked that minimal scrolling is required on the home page. They also particularly liked the *Quick links* and *Jump to top* functions, as they offered straightforward navigation. In addition, they stated that content written in an easily understandable language and arranged in a structured manner added value to the website and improved the overall user experience.

**Positive accessibility features of the website.**

Consistent layout and navigation:

“I like the uniform layout and colour theme. The text all have the same size throughout which is good as I did not have to change my magnification level often. The left-aligned texts make it easier to read as well. The texts on the Wordpress design can be easily missed on magnified view as they are not all properly aligned.” [P4, 62-year-old cone-rod dystrophy patient]

“The zig-zag arrangement of icons in the Wordpress design can be bad for accessibility.” [P5, 40-year-old patient with aniridia]

Structured information hierarchy with a clear description of links:

“The headings and subheadings of each page are very clear. Each heading describes exactly what is written.” [P8, 45-year-old unaffected individual]

“I found it easy to navigate through the different headings for each page using the ‘Insert-F6’ function on JAWS which lists all the headings.” [P9, 70-year-old patient with cone-rod dystrophy]

“I like the bullet point arrangement which made it easy to read. The targets of the links are also very clear with accurate descriptions.” [P5, 40-year-old patient with aniridia]

“The Wikipedia articles have too long paragraphs; having bullet points will make it easier to read.” [P1, 53-year-old unaffected father of a patient with type II Usher syndrome]

“The links are relevant in gene.vision and work well. There are too many links to read with a screen reader on Wikipedia.” [P6, 37-year-old patient with retinitis pigmentosa]

Adaptability to different assistive software:

“The website works really well and is really fluid when using Voiceover on my iPhone.” [P3, 41-year-old patient using iOS Voiceover with retinitis pigmentosa]

“I am still able to see the whole page at 200% magnification*.*” [P5, 40-year-old patient using Windows magnifier with aniridia]

“The website works well with my Zoomtext settings.” [P4, 62-year-old patient using ZoomText 2020 with cone-rod dystrophy patient]

“I found the website easy to understand and JAWS friendly.” [P9, 70-year-old patient using JAWS with cone-rod dystrophy]

Simple home page with easy navigation:

“The home page is all on one page, which I like. No sliding or scrolling required.” [P1, 53-year-old unaffected father of a patient with type II Usher syndrome]

“The drop-down menu at the top works well and as expected. The simpler the navigation the better. Gene.vision is easier to navigate through compared to Wikipedia.” [P3, 41-year-old patient with retinitis pigmentosa]

“I like the drop-down menu as less scrolling is involved. I really like the ‘Jump to top’ button to navigate around when reading.” [P4, 62-year-old patient with cone-rod dystrophy]

“I really like the table of contents at the top as I can go straight into any topic that interests me.” [P2, 52-year-old unaffected mother of a patient with type II Usher syndrome]

Readable content:

“The gene.vision page speaks to a patient or a family member. I think it addresses the issues they want to know about. The content is not too detailed, digestible and easy to understand.” [P8, 45-year-old unaffected individual]

“The language used in gene.vision is good and at a good level, simple enough if you have just been diagnosed. It works well and simpler than the Wikipedia page with Voiceover.” [P3, 41-year-old patient with retinitis pigmentosa]

“Gene.vision is easier to read and have a softer tone than Wikipedia. The layout is more user friendly as well*.*” [P6, 37-year-old patient with retinitis pigmentosa]

Although unrelated to accessibility, our participants pointed out that content quality was equally important to attract users. With respect to the prototype, they felt that content related to research, support available, practical advice, and links to charities were incentives for repeated visits. They also suggested that knowing the information is coming from a reliable source will make the website more trustworthy. Quotes from participants that illustrate this point are as follows:

Reading stuff that is tough or difficult to absorb, it is good to have links to support. I also like the practical advice section with further links to topics that are important to me as a mother.

P2, 52-year-old unaffected mother of a patient with type II Usher syndrome

The content is pitched about right with links to more scientific content or papers available for doctors or patients who wanted to find out even more and look at the research themselves. It would be good to know that the website is definitely accurate if endorsed by an approved authority.

An excerpt from the patient focus group

On the other hand, our participants also identified some features on our website that impaired its usability not only for people with visual impairment but also for individuals with unimpaired vision (Textbox 4). It was highlighted to us that having good contrast is essential to accessibility. Certain aspects of our prototype, especially the navigation bar and the accompanying search box (Figure 1), had poor contrast and proved difficult for some participants to identify. One participant also pointed out that links should be underlined as default for easy identification (Figure 4). Furthermore, we found that implementing dynamic content such as an automatic timed *carousel* function (Multimedia Appendix 2) and large white spaces may frustrate and confuse screen reader and magnifier users. Having predictable navigation on a website is also crucial for accessibility. Most users are familiar with how popular search engines (Google, Bing, etc) work and they would expect similar functions when using other search boxes. This was proven when our participants found the search results page on our prototype confusing (Figure 2).

**Negative accessibility features of the website.**

Poor chromatic and luminance contrast:

“The navigation bar is not easily found due to low contrast. I could not identify the search box on the navigation bar as it is very faint. When my pointer is at the genetics menu, it did not ‘light up’ very well to indicate that I am there. I find it difficult to tell if it is a link without hovering over the text with my mouse in gene.vision.” [P4, 62-year-old patient with cone-rod dystrophy]

“The navigation bar is difficult to find. Grey is not visible, making it hard to see at first.” [P8, 45-year-old unaffected individual]

“The ‘for patients’ and ‘for doctors’ tabs are not obvious due to poor contrast. The search box and search button could be more visible.” [P5, 40-year-old patient with aniridia]

Dynamic content and large white spaces:

“I don’t like the distraction of a carousel, especially one that I can’t manually control. It doesn’t allow me enough time to read its content.” [P5, 40-year-old patient with aniridia]

“There are a lot of white space on the right side of the gene.vision page, magnifier users may get lost. The spacing is better in portrait mode on an iPad. It needs a more responsive layout.” [P6, 37-year-old patient with retinitis pigmentosa]

Unpredictable search behavior:

It is difficult to find links to click on from the search results page. The ‘Page title matches’ and ‘Page text matches’ are confusing. [P8, 45-year-old unaffected individual]

“How would I use the top search box to get all the pages including the word aniridia? I have to go down to the ‘containing’ bit.” [P5, 40-year-old patient with aniridia]

#### Clinician Feedback and Recommendations

All the health care professionals who tested our website felt that the information provided was comprehensive and useful. They particularly liked the *Quick links* function, which enabled them to skip to the section of interest instantly. Both consultant and trainee ophthalmologists found the information on current research very helpful, as the listed investigational therapies were explained in a simple yet detailed manner, enabling them to gain a basic understanding without having to read multiple papers. They also liked that they had quick access to scientific abstracts of seminal papers through the hyperlinks provided in each condition page. Finally, the *Information and support* menu and links to condition-specific charities were highly welcomed by all, regardless of discipline or subspecialty, as such queries are commonly posed by patients and their families in clinics. In addition, the involved health care professionals also provided some suggestions to further enhance the usability of our website. These and the improvements we have introduced to our current iteration are summarized in Textbox 5.

**Suggested features that we have added to the current version of the Gene.Vision website.**

Suggestions

- Changing the *Overview* section of each condition to a table format for easy reference in a busy clinic
- Having a dedicated page explaining about clinical genetic testing so that clinicians can gain a basic understanding of the various sequencing techniques that are currently available
- Knowing the associated phenotypes of a specific gene can be helpful in directing further clinical management (ie, if there are any associated systemic features)
- Referral centers for specialist clinics and/or treatment should be listed where appropriate to improve practicality (eg, centers in the United Kingdom that administer a recently approved retinal gene therapy called Luxturna [24])

Actions

- The *Overview* section for each condition has been changed to a comprehensive table format
- A page on clinical genetic testing has been added to the site, providing a brief overview of the different types of sequencing methods and also covering the benefits, limitations and potential ethical concerns associated with genetic testing
- A feature called *gene cards* has been added (Multimedia Appendix 6), covering some of the more common genes listed on the Great Ormond Street Hospital for Children oculome panel [25]
- Specialist clinics for very rare conditions (eg, Bardet-Biedl syndrome) and referral centers for Luxturna treatment in the United Kingdom are listed in their associated pages

## Discussion

### Principal Findings

We evaluated the initial designs of a health information website focused on rare genetic eye disorders for patients, relatives, and health care professionals. Early development centered on meeting the user requirements of our patients, most of whom had visual impairment or blindness. We wanted to identify features that optimized or impaired accessibility for users with visual impairment to ensure that this vulnerable group was not excluded from information that could potentially benefit them and to improve overall user uptake.

From this testing session, we identified the following features that will enhance accessibility and usability for users with visual impairment:

- Consistent website layout and fonts.
- Structured information hierarchy with a clear description of links.
- Good chromatic and luminance contrast.
- Simple home page with consistent, predictable, and easy navigation.
- Readable content (appropriate to the intended audience).
- Adaptability to various assistive software and mobile devices.
- Avoidance of dynamic content and large *white spaces*.

In addition to good accessibility, content quality and reliability were highly rated as potential factors that may influence user traffic. They also suggested that having an authority endorsing a website will increase its trustworthiness.

On the basis of the above feedback, we updated our website by changing the features that negatively impacted accessibility for our participants. These changes were as follows:

- Improving the contrast of the navigation bar.
- Hyperlinks are bolded and underlined as default.
- Making the search box larger.
- Having a more conventional search behavior with an autocomplete function.
- Incorporating the *breadcrumb* functionality so that users can go back to the previous page that they were visiting.

We have kept features that our participants found useful as listed under *positive accessibility features of the website* in the Results section. The screen recording of the updated website is in Multimedia Appendix 6. The navigation bar and home page menu have also been updated after adding more content to the current version.

### Limitations

Our study was limited by a small cohort of participants, none of whom were Braille keyboard users. Most of the participants were technologically adept (8 participants had at least basic digital skills) and belonged to the middle age bracket. Therefore, for future testing sessions, users with lower digital skills and a broader age spectrum inclusive of younger and older patients are recommended. This will consequently provide a wider opinion about web-based accessibility for people with visual impairment.

### Comparison With Previous Work

The web-based behaviors of users with visual impairment differ significantly from those of individuals with full sight. Users with visual impairment tend to employ tactics to navigate around websites more efficiently. These include probing (getting a glimpse of a page by traversing it in a sequential fashion or by jumping between headings) [26], gambling scanning (skipping a determined number of lines until bumping into content that draws their interest) [27], and memorizing the amount of links that need to be skipped to get to the main content [28]. Some users may listen to the content sequentially from the beginning (exhaustive scanning), especially when visiting unfamiliar pages or sites [29].

Web accessibility for users with visual impairment can be improved by understanding the barriers they commonly encounter through usability testing and leveraging on the aforementioned navigational strategies. By having a consistent and well-structured information layout with clear headings, users were able to quickly orientate themselves without getting lost in a sea of texts [30]. This was further enhanced on our website by the *Quick links and Jump to top* functions where participants were able to navigate directly to the topic of interest without scrolling. The *Jump to top* function also served as a *shelter* if users lost their orientation within the page as they can go back to the beginning of the page instantly, which is a tactic some users employ when they are lost [30]. Clear description of links is also equally important for effective navigation, particularly for screen reader users [31].

Users with visual impairment may feel frustrated and give up on exploring a particular website further if they are faced with overwhelming situations such as a large number of search results or taking too many steps to complete a transaction [30]. The search behavior of our prototype is a prime example (Figure 2), where the search term *aniridia* yielded multiple results under the heading *page title matches* and *page text matches* with confusing excerpts. Hence, we have updated our search behavior to reflect conventional search engines and also incorporate an autocomplete function as many medical terms are not in common usage, which can make it challenging for lay users to spell accurately.

It is believed that many websites are not accessible to users with disability because developers fear that optimizing accessibility can be costly and may affect the attractiveness of a website to sighted users [32,33]. However, there is an abundance of evidence showing that usability problems are shared regardless of visual disability and everyone stands to benefit from good accessibility [34-36]. Participants with and without visual impairment in our testing session demanded similar features, such as having a simplistic design without any dynamic content. A simple and well-designed page has fewer elements to navigate and requires less time to load [31]. Furthermore, carousels cannot be manipulated with keyboards, which can lead to frustration among screen reader users. Other features such as easily readable fonts and good contrast are also crucial and are covered in the WCAG guidelines [7].

Another factor taken into consideration when designing the website was the increasing popularity of mainstream mobile devices such as smartphones and tablet computers, both among those with and without visual impairment [37]. Thus, having a *responsive* website skin that adapts to different screen sizes has been highlighted by our participants as an important accessibility feature. This will have a direct and significant impact on everyone, as nearly three-fourth of internet users are predicted to access the web solely through smartphones by 2025 [38].

This study also demonstrated the benefits of designing a website with a user-centric approach that was previously outlined by Abelse et al [39] and further emphasized in the design principles of the NHS Digital Service Manual [40]. By understanding the challenges and requirements of users, we can then create solutions and further refine them based on feedback. This is assisted by having various prototype versions so that multiple features can be tested in a single session. For example, we were able to quickly identify features that optimized or affected accessibility for those with visual impairment by having our participants test 2 home page prototypes.

Testing with target users can also provide developers with a more accurate idea of subjective features such as readability of the content and website navigation. Although most of the accessibility characteristics identified by our participants were covered by the WCAG guidelines, such parameters are often difficult or even impossible to assess with automated tools. Even if a website conforms to the WCAG guidelines, it may not ensure good accessibility as pointed out by Power et al [6]. Their study found that the WCAG 2.0 guideline only covered half of the user problems encountered by their cohort of screen reader users. Furthermore, they reported that user problems still existed despite the implementation of some of the recommendations. The authors suggested that enhancing website accessibility should shift from a traditional problem-based approach to gathering user data from testing sessions with real-world users, which is the approach we took.

Content quality is equally crucial in attracting users [39]. As a website aiming at patients and families affected by rare genetic eye disorders, we believed that having information on *soft* content, such as practical advice, available support, and charities were important to our target audience. This resonated well with our cohort and one of them, a mother of a patient affected by type II Usher syndrome (P2) stated that knowing where to look for support and learning about practical tips that will help her son’s daily living was more important as these were issues that “kept me up at night.” Litzkendorf et al [41] reported similar findings in their study where patients with rare diseases were interviewed about their information needs and acquisition. The main themes that were lacking in rare disease websites were current research, practical tips to cope with a condition, genetic counseling and family planning options, social and educational support, and the ability to connect with similar people.

The lack of exposure to rare diseases among clinicians has often led to delayed diagnoses and incorrect care for many patients. Recent surveys of primary and secondary care physicians in Belgium and Spain have identified a huge demand for information on rare diseases, particularly on genetic screening and counseling, investigational therapies, specialist referral centers, and locating reliable resources [42,43]. Although information portals for rare diseases such as Orphanet already exist, health care professionals who tested our website believed that Gene.Vision is highly relevant as it offers more in-depth information on genetic eye disorders and covers the available social support for individuals with visual impairment in the United Kingdom. Furthermore, as genomic sequencing becomes more accessible and affordable in the United Kingdom, nonmolecular ophthalmologists, with appropriate training and guidance, are encouraged to undertake genetic testing for their patients to aid diagnosis, management, and trial enrollment [44]. Once a molecular diagnosis is received, the *gene card* feature on our website supplements this by providing a quick overview of the associated phenotype or phenotypes and information on current research.

### Conclusions

To make a website accessible to users with visual impairment, attention should be focused on making simple, well-designed pages with consistent layout and information structure, good contrast, and simple navigation, all of which will directly improve the overall user experience. Although most of these features are part of the WCAG 2.0 and 2.1 recommendations, usability testing with real-world users should be conducted as well as examination by professional testers and use of automated web accessibility evaluation tools. More generally, a website’s design and content should be developed with the input of target users from the earliest stages to ensure that it meets their needs. Many of these steps can be implemented easily and will help in search engine optimization.

## Appendix

Multimedia Appendix 1 Screen recording of the Gene.Vision prototype homepage based on the Wikimedia system.

Multimedia Appendix 2 Screen recording of the Gene.Vision prototype homepage designed on WordPress.

Multimedia Appendix 3 Screen recording demonstrating the “Quick links” and “Jump to top” functions in the condition pages of the prototype.

Multimedia Appendix 4 Survey form for participants of the usability testing session.

Multimedia Appendix 5 Full results of the pretesting survey.

Multimedia Appendix 6 Screen recording of the updated Gene.Vision website based on patient and clinician feedback from the usability testing session. The RPE65 gene card is demonstrated here.

## Abbreviations

JAWS: Job Access With Speech
NHS: National Health Service
WAGR: Wilms tumor, aniridia, genitourinary anomalies, and range of developmental delay
WCAG: Web Content Accessibility Guidelines

## References

[1] (2017). Being disabled in Britain: a journey less equal. Equality and Human Rights Commission.

[2] (2019). Access and Inclusion in 2018: Consumers' experiences in communications markets. Office of Communications.

[3] Wu Y, Lindsay S, Cable J, Jones R, Evans L, Xianghua X (2018). Digital Media Usage of Sensory Impaired Users in Wales. Swansea University.

[4] Seixas Pereira L, Archambault D (2017). Web widgets barriers for visually impaired users. Stud Health Technol Inform.

[5] Royal National Institute of Blind People (RNIB) (2014). The economic impact of sight loss and blindness in the UK adult population, 2013. Deloitte Access Economic.

[6] Power C, Freire A, Petrie H, Swallow D (2012). Guidelines are only half of the story: accessibility problems encountered by blind users on the web. Proceedings of the SIGCHI Conference on Human Factors in Computing Systems.

[7] Kirkpatrick A, Connor J, Campbell A, Cooper M (2018). Web Content Accessibility Guidelines (WCAG) 2.1. World Wide Web Consortium.

[8] Government Digital Service (2018). Understanding new accessibility requirements for public sector bodies. Government of the United Kingdom.

[9] (2019). Web Accessibility. European Commission.

[10] Christopherson R (2018). 'Web Accessibility Guidelines' turn 10 but still less than 10% of sites are accessible. AbilityNet.

[11] (2019). Design and build digital services for the NHS. National Health Service (NHS).

[12] Rare Disease UK (2018). What is a rare disease?. Genetic Alliance UK.

[13] Pauer F, Litzkendorf S, Göbel J, Storf H, Zeidler J, Graf von der Schulenburg J (2017). Rare diseases on the internet: an assessment of the quality of online information. J Med Internet Res.

[14] Lüchtenberg M, Kuhli-Hattenbach C, Sinangin Y, Ohrloff C, Schalnus R (2008). Accessibility of health information on the internet to the visually impaired user. Ophthalmologica.

[15] Liew G, Michaelides M, Bunce C (2014). A comparison of the causes of blindness certifications in England and Wales in working age adults (16-64 years), 1999-2000 with 2009-2010. BMJ Open.

[16] Gilbert C, Foster A (2001). Childhood blindness in the context of VISION 2020--the right to sight. Bull World Health Organ.

[17] Duran M (2017). Accessibility in government: What we found when we tested tools on the world's least-accessible webpage. Government of the United Kingdom.

[18] (2019). How to make digital services accessible. National Health Service.

[19] Yeong JL, Thomas P, Buller J, Moosajee M (2019). A resource for patients and doctors about rare genetic eye disorders. Gene Vision.

[20] United Kingdom Government Digital Service, United Kingdom Government Cabinet Office (2014). Policy paper: Government Digital Inclusion Strategy. Government of the United Kingdom.

[21] Heilman JM, West AG (2015). Wikipedia and medicine: quantifying readership, editors, and the significance of natural language. J Med Internet Res.

[22] Laurent MR, Vickers TJ (2009). Seeking health information online: does Wikipedia matter?. J Am Med Inform Assoc.

[23] (2020). Wikipedia:Manual of Style/Accessibility. Wikipedia.

[24] Russell S, Bennett J, Wellman JA, Chung DC, Yu Z, Tillman A, Wittes J, Pappas J, Elci O, McCague S, Cross D, Marshall KA, Walshire J, Kehoe TL, Reichert H, Davis M, Raffini L, George LA, Hudson FP, Dingfield L, Zhu X, Haller JA, Sohn EH, Mahajan VB, Pfeifer W, Weckmann M, Johnson C, Gewaily D, Drack A, Stone E, Wachtel K, Simonelli F, Leroy BP, Wright JF, High KA, Maguire AM (2017). Efficacy and safety of voretigene neparvovec (AAV2-hRPE65v2) in patients with RPE65-mediated inherited retinal dystrophy: a randomised, controlled, open-label, phase 3 trial. Lancet.

[25] Patel A, Hayward JD, Tailor V, Nyanhete R, Ahlfors H, Gabriel C, Jannini TB, Abbou-Rayyah Y, Henderson R, Nischal KK, Islam L, Bitner-Glindzicz M, Hurst J, Valdivia LE, Zanolli M, Moosajee M, Brookes J, Papadopoulos M, Khaw PT, Cullup T, Jenkins L, Dahlmann-Noor A, Sowden JC (2019). The Oculome panel test: next-generation sequencing to diagnose a diverse range of genetic developmental eye disorders. Ophthalmology.

[26] Goble C, Harper S, Stevens R (2000). The travails of visually impaired web travellers. Proceedings of the eleventh ACM on Hypertext and hypermedia.

[27] Takagi H, Saito S, Fukuda K, Asakawa C (2007). Analysis of navigability of web applications for improving blind usability. ACM Trans Comput Hum Interact.

[28] Yesilada Y, Stevens R, Harper S, Goble C (2007). Evaluating DANTE: semantic transcoding for visually disabled users. ACM Trans Comput Hum Interact.

[29] WebAIM (2012). Screen reader user survey #4. WebAIM.

[30] Vigo M, Harper S (2013). Coping tactics employed by visually disabled users on the web. Int J Hum Comp Stud.

[31] Kurt S (2018). Moving toward a universally accessible web: web accessibility and education. Assist Technol.

[32] Ellcessor E (2014). Web Accessibility Myths as Negotiated Industrial Lore. Crit Stud Media Commun.

[33] Petrie H, Hamilton F, King N (2004). Tension, what tension? Website accessibility and visual design. Proceedings of the 2004 international cross-disciplinary workshop on Web accessibility (W4A).

[34] Petrie H, Kheir O (2007). The relationship between accessibility and usability of websites. Proceedings of the SIGCHI Conference on Human Factors in Computing Systems.

[35] Yesilada Y, Brajnik G, Vigo M, Harper S (2013). Exploring perceptions of web accessibility: a survey approach. Behav Inf Technol.

[36] Schmutz S, Sonderegger A, Sauer J (2017). Implementing recommendations from web accessibility guidelines: a comparative study of nondisabled users and users with visual impairments. Hum Factors.

[37] Martiniello N, Eisenbarth W, Lehane C, Johnson A, Wittich W (2019). Exploring the use of smartphones and tablets among people with visual impairments: are mainstream devices replacing the use of traditional visual aids?. Assist Technol.

[38] Handley L (2019). Nearly three quarters of the world will use just their smartphones to access the internet by 2025. CNBC.

[39] Abelse EG, Domas White M, Hahn K (1998). A user‐based design process for web sites. Internet Res.

[40] (2018). Design principles. National Health Service.

[41] Litzkendorf S, Babac A, Rosenfeldt D, Schauer F, Hartz T, Lührs V, Graf von der Schulenburg JM, Frank M (2016). Information needs of people with rare diseases-what information do patients and their relatives require. J Rare Dis Diagn Ther.

[42] Ramalle-Gómara E, Domínguez-Garrido E, Gómez-Eguílaz M, Marzo-Sola ME, Ramón-Trapero JL, Gil-de-Gómez J (2020). Education and information needs for physicians about rare diseases in Spain. Orphanet J Rare Dis.

[43] Vandeborne L, van Overbeeke E, Dooms M, De Beleyr B, Huys I (2019). Information needs of physicians regarding the diagnosis of rare diseases: a questionnaire-based study in Belgium. Orphanet J Rare Dis.

[44] Black GC, MacEwen C, Lotery AJ (2020). The integration of genomics into clinical ophthalmic services in the UK. Eye (Lond).

