# Correction: A Newly Developed Web-Based Resource on Genetic Eye Disorders for Users With Visual Impairment (Gene.Vision): Usability Study

**DOI:** 10.2196/27330

**Published:** 2021-01-25

**Authors:** Jian Lee Yeong, Peter Thomas, James Buller, Mariya Moosajee

**Affiliations:** 1 Institute of Ophthalmology University College London London United Kingdom; 2 Moorfields Eye Hospital NHS Foundation Trust London United Kingdom; 3 Aniridia Network Sheffield United Kingdom; 4 Great Ormond Street Hospital for Children NHS Foundation Trust London United Kingdom

In “A Newly Developed Web-Based Resource on Genetic Eye Disorders for Users With Visual Impairment (Gene.Vision): Usability Study” (J Med Internet Res 2021;23(1):e19151) the authors noted one error.

In the originally published article, additional text was inadvertently included at end of the Background section of the Abstract. It read as follows:

Despite the introduction of the Web Content Accessibility Guidelines and legislations, many websites remain poorly accessible to users with disability, especially those with visual impairment, as the internet has become a more visually complex environment. With increasing reliance on the internet and almost 2 million people in the United Kingdom being affected by vision loss, it is important that they are not overlooked when developing web-based materials. A significant proportion of those affected have irreversible vision loss due to rare genetic eye disorders, and many of them use the internet as a primary source of information for their conditions. However, access to high-quality web-based health information with an inclusive design remains a challenge for many. We have developed a new web-based resource for genetic eye disorders called Gene.Vision that aims to provide a holistic guide for patients, relatives, and health care professionals. by sight loss, it is important that they are not overlooked when developing web-based materials. A significant proportion of those affected have irreversible sight loss due to rare genetic eye disorders, and many of them use the internet as a primary source of information for their conditions. However, access to high-quality web-based health information with an inclusive design remains a challenge for many.

This section has been revised to:

Despite the introduction of the Web Content Accessibility Guidelines and legislations, many websites remain poorly accessible to users with disability, especially those with visual impairment, as the internet has become a more visually complex environment. With increasing reliance on the internet and almost 2 million people in the United Kingdom being affected by vision loss, it is important that they are not overlooked when developing web-based materials. A significant proportion of those affected have irreversible vision loss due to rare genetic eye disorders, and many of them use the internet as a primary source of information for their conditions. However, access to high-quality web-based health information with an inclusive design remains a challenge for many. We have developed a new web-based resource for genetic eye disorders called Gene.Vision that aims to provide a holistic guide for patients, relatives, and health care professionals.

The correction will appear in the online version of the paper on the JMIR Publications website on January 25, 2021, together with the publication of this correction notice. Because this was made after submission to PubMed, PubMed Central, and other full-text repositories, the corrected article has also been resubmitted to those repositories.

