# Supplementary material for: A Newly Developed Web-Based Resource on Genetic Eye Disorders for Users With Visual Impairment (Gene.Vision): Usability Study
Source: J Med Internet Res. 2021 Jan 20;23(1):e19151. doi: 10.2196/19151 (PMC7857953; doi:10.2196/19151)
Supplement: Multimedia Appendix 4 [file jmir_v23i1e19151_app4.pdf]

<u>Focus group meeting (11/11/19)</u>

Facilitator: Participant:

Time: 1015-1030 (15 minutes)

Pre-testing survey

1) What is your age?

2) What is your employment status?

☐Employed

What is your occupation?

☐Unemployed

☐Retired

What was your previous occupation?

3) Do you use the Internet?

☐Yes (proceed to question 5)

☐No (proceed to question 4)

4) What is the main reason?

☐Limited skills (limited ability to use the Internet and online services)

☐Limited access (no or poor connectivity to the Internet in your area)

☐Lack of confidence (fear of online crime, do not know where to start)

☐Lack of motivation (do not see why the Internet could be relevant or helpful)

5) Do you use any of the following devices? (Please select any that applies)

☐Computer (desktop/laptop)

☐Smartphones (iPhones, Android, Windows mobile)

☐Tablets (iPad, Android, Kindle)

6) What do you mainly use it for? (Please select only one option for each section where applicable)

(a) Computer

☐Online activities (using the Internet, e-mails, banking, social media, shopping, Netflix, YouTube, Spotify, iTunes etc)

☐Offline activities

(b) Smartphones

☐Online activities (using the Internet, e-mails, banking, social media, shopping, Netflix, YouTube, Spotify, iTunes, e-books, audio books, podcasts etc)

☐Offline activities (phone calls, messaging, taking photos)

(c) Tablets

☐Online activities (using the Internet, e-mails, banking, social media, shopping, Netflix, YouTube, Spotify, iTunes, e-books, audio books, podcasts etc)

☐Offline activities (taking photos, games)

7) Do you use any assistive technology or visual aids when using any of your devices?

☐Yes (proceed to question 8)

☐No (proceed to question 9)

8) What are software(s) or visual aid(s) do you use? Please specify.

9) Of the device(s) stated, which one do you use most often to access the Internet?

10) What do you mainly use the Internet for? (You can select up to three options)

☐Communications (e-mails, Facebook, Instagram, Twitter, Skype etc)

☐News and learning (includes e-books, audio books, podcasts etc)

☐Entertainment (includes Netflix, BBC iPlayer, YouTube, Vimeo, Spotify, iTunes, gaming etc)

☐Online shopping (Amazon, eBay etc)

☐Banking and utilities

☐Research on a topic of interest (includes looking at articles, videos, images on the topic)

☐Other. Please specify:

11) How confident are you in using the Internet? (Please only select one option based on the answers given in question 10)

☐Not at all confident (Do not know how to use the Internet at all or only able to do above tasks with full guidance)

☐Slightly confident (Able to perform the above tasks with some guidance)

☐Somewhat confident (Able to perform the above tasks well independently, but do not use the Internet for other tasks; "Task-specific")

☐Very confident (Able to perform the above tasks well and also explore other functions of the Internet; "Basic digital skills")

☐Extremely confident (An expert. Have background in computing, able to write codes and design content)

12) Do you or any of your family members suffer from an inherited eye disorder? What is the diagnosis?

☐I am a patient

What is your diagnosis?

☐I am a family member

13) How do you normally find information about your condition? (Please select any that applies)

☐Asking the doctor in clinic

☐Family and friends

☐Speaking (phone call or speaking face-to-face) to a representative from a charity (Retina UK, Aniridia Network, RNIB etc)

☐Online research (Please specify where. This includes charity websites)

☐Other

☐Not really interested

14) Do you think your source(s) has provided sufficient amount of information for you?

☐Yes (end of survey)

☐No (proceed to question 15)

15) What would you like to know more? (Please state only one aspect)

